# Assessment of blinding to treatment allocation in studies of a cannabis-based medicine (Sativex®) in people with multiple sclerosis: a new approach

**DOI:** 10.1186/1745-6215-13-189

**Published:** 2012-10-09

**Authors:** Stephen Wright, Paul Duncombe, Douglas G Altman

**Affiliations:** 1 GW Pharma, Ltd, Porton Down Science Park, Salisbury, Wiltshire, SP4 0JQ, UK; 2 Centre for Statistics in Medicine, University of Oxford, Oxford, UK

**Keywords:** Treatment allocation, Double-blind, Sativex®

## Abstract

**Background:**

Maintenance of the blind-to-treatment allocation is one of the most important means of avoiding bias in randomised controlled clinical trials. Commonly used methodologies to determine whether patients have become unblinded to treatment allocation are imperfect. This may be of particular concern in studies where outcomes are patient-reported, and with products which have a characteristic adverse event profile. We report the results of an evidence-based statistical approach to exploring the possible impact of unblinding to a cannabis-based medicine (Sativex®) in people with muscle spasticity due to multiple sclerosis.

**Methods:**

All 666 patients included in three Phase III placebo-controlled studies were included in this analysis. The relationship between factors that might permit patients to identify their treatment allocation and the effect of treatment on the self-reported primary outcome measure was investigated using a general linear model where the dependent variable was the change from baseline in patient self-reported spasticity severity, and the various possible explanatory factors were regarded as fixed factors in the model.

**Results:**

There was no significant relationship between the effect of Sativex® on spasticity and the prior use of cannabis or the incidence of ‘typical’ adverse events. Nor was there any significant relationship between the prior use of cannabis and the incidence of ‘typical’ adverse events, nor between prior use of cannabis and dose of Sativex®.

**Conclusions:**

There is no evidence to suggest that there was widespread unblinding to treatment allocation in these three studies. If any patients did become unblinded, then there is no evidence that this led to bias in the assessment of the treatment difference between Sativex® and Placebo for efficacy, adverse events or study drug dosing. This methodology may be suitable for assessment of the integrity of the blind in other randomized clinical trials

## Background

In the planning, execution and analysis of clinical trials, bias may be introduced by a number of factors, one of which is that patients or investigators in the study have become aware of their treatment allocation [1,2]. Randomised trials are often designed as “double-blind” in order to help minimize this source of bias. Lack of blinding is a particular concern for trials with important subjective outcomes.

When blinding is not possible the trial is called “open-label”. Unblinding within a randomised controlled trial is unlikely to occur for all patients, in which case it would be an open-label comparison. When the blind is broken the patient (or investigator) is certain about the allocation of treatment. Much more likely is that the blind is broken for a subset of participants or that there is a degree of belief (but not certainty) about treatment allocation, in which case the extent of potential bias introduced is unclear. When the primary outcome of the study is an objective measurement, unaffected by the patient’s or investigator’s knowledge of the treatment allocation, there may be few consequences for the assessment of efficacy, although bias may then affect the assessment of adverse effects [3]. On the other hand, when the outcome measure is a subjective report by the patient or by the investigator, then both the therapeutic effect and the adverse event profile may be influenced if the patient is aware which treatment they are taking [4]. Either way, assessment of the risk/benefit may be subject to bias.

The maintenance of the blind to treatment allocation could be compromised in several ways. These include:

1. The occurrence of particular adverse events, combined with the information given to the patient on the known adverse event profile, might enable some patients to deduce they are on a particular study medication. This possibility may be exacerbated for patients with previous experience of similar compounds, using their reactions to these compounds to influence which study medication they think they are receiving.

2. The efficacy of a study medication may suggest which treatment is being received. This possibility is more likely when the comparator is a placebo or a known ineffective compound, and the active medication is effective.

The second of these causes of unblinding is not generally a serious concern, since if a patient believes that they are on the active study medication as a result of efficacy then the presence of unblinding is effectively a surrogate endpoint for efficacy. Consequently, an analysis of unblinding that shows more correct ‘guesses’ in the group with the better outcome is not necessarily an indication that the blind was broken [3,5-7] .

Despite this, the most widely used methodology to investigate the success of blinding within a clinical study is to ask the patient at some point or points during the study to guess whether they are taking the active or the placebo preparation. The proportion of correct guesses is then compared between the treatment groups. But testing trial participants (or their clinicians) for blindness at the end of a trial cannot distinguish the failure of blinding tactics from their correct guesses about which treatment was received, based on their experiences of pharmacodynamics, side-effects and trial outcomes [8].

Nonetheless, the possibility of a break in the blind is sometimes a real concern, especially in trials with a subjectively assessed outcome. This question arose in conjunction with a group of trials of a medicine to treat patients with multiple sclerosis.

Sativex® (GW Pharma, Ltd, Salisbury, Wiltshire, UK), is a recently authorised medicine used for the treatment of spasticity in people with multiple sclerosis, and is derived from an extract of the cannabis plant. Its principal components are the two major cannabinoids, delta-9-tetrahydrocannabinol (THC) and cannabidiol (CBD). In order to avoid the extensive first pass metabolism associated with the oral use of cannabinoids, it is taken as a 100 microlitre spray administered to the sub-lingual and buccal mucosal surfaces. Each spray contains 2.7 mg THC and 2.5 mg CBD. THC is the psychoactive component of Sativex®, and is responsible for the psychoactivity which recreational users of cannabis seek; CBD, on the other hand, is not psychoactive, and has been shown to modulate some aspects of the pharmacodynamics of THC. Both components have a distinct pharmacology, and together have been shown to relieve spasticity in people with multiple sclerosis [9-11].

With any medicine containing psychoactive components, the presence of central nervous system side effects may lead the patient into guessing that they are taking the active medicine. Also, if the patient has previously experienced the psychoactive effects of cannabis, there is a possibility that they may ‘recognise’ the active study medication. This is a phenomenon that may apply to a range of medicines with a typical CNS side effect profile, and not only to a cannabis-based medicine.

To explore whether there was evidence of unblinding in its key efficacy studies, we combined data from three randomised, parallel-group, placebo-controlled studies of Sativex® in the treatment of spasticity. There had not in fact been an attempt to assess the success of blinding during the conduct of those trials. We examined whether the prior use of cannabis or the experience of the most typical central nervous system adverse events were associated with its anti-spasticity efficacy. We also investigated whether prior use of cannabis predicted the dose of Sativex® to which patients titrated, or their experience of any of the most typical CNS adverse events.

There is a consensus, eloquently expressed in the Cochrane review of anti-spasticity agents in multiple sclerosis [12], that an acceptable efficacy endpoint ‘must reflect the patient’s daily experience of their spasticity’. This opinion, taken together with the inadequacy of the Ashworth Scale [13], means that a patient-reported outcome measure is the preferred outcome measure in the assessment of spasticity. The numerical rating scale, preferred as the primary outcome measure in studies of pain, has been validated in the assessment of spasticity due to multiple sclerosis [14,15].

## Methods

The analyses of efficacy data used the combined intention to treat (ITT) (Full Analysis) populations from three randomised parallel group double-blind trials comparing Sativex® with placebo. The results of each of these studies have been separately published, and we will not describe them further here [9-11]. Each study included a similar population of patients, each study was of similar design, and each study used a patient-reported spasticity severity score as the primary assessment of the severity of spasticity. In one study [9], the patients assessed their spasticity on a 0 to 100 mm visual analogue scale. These data were converted to a 0 to 10 scale by dividing each individual observation by 10. Both the other studies [10,11] used a patient-reported 0 to 10 Numeric Rating Scale (NRS) on which 0 represented the complete absence of spasticity and 10 the worst ever spasticity.

The three studies were combined in a pre-planned meta-analysis [16] which showed no statistically significant study-by-treatment interaction (*P* >0.1) or heterogeneity of variance (*P* >0.1). Overall, the meta-analysis showed Sativex® to be superior to placebo as assessed by the change in spasticity severity according to the NRS at the end of the study (treatment difference of 0.32 units, SE diff = 0.145; 95% CI −0.61 to −0.04; *P* = 0.026). Secondary measures of anti-spasticity effect included a 30% responder analysis (OR 1.62; 95% CI 1.15 to 2.28; *P* = 0.0073), a Patient Global Impression of Change (OR 1.66; 95% CI 1.16 to 2.9: *P* = 0.0036) and an Ashworth Score (no significant difference between treatments).

The analyses of adverse events (AEs) and dose of test medication used study participants who received at least one dose of study medication (the combined safety populations). In all of the analyses, imputation for missing values was not done. Consequently, cases with missing values were removed from the affected analyses. In particular, this includes missing values for baseline covariates (12 participants) and for prior exposure to cannabis (1 participant).

The spasticity data were analysed using a general linear model. The dependent variable was the change from baseline in mean spasticity assessment. Fixed factors considered in the models were treatment group (Sativex®/placebo) (TG), prior use of cannabis (Yes/No) (PC), study (ST), and the interaction terms TG*PC, TG*ST, PC*ST, TG*PC*ST. Mean baseline spasticity (BS), measured on a continuous 0 to 10 scale, was included as a covariate in all models. Models containing the following terms were fitted sequentially; with each subsequent model being considered only if the term to be removed from the current model did not contribute significantly (*P* <0.05):

Model 1. BS, ST, PC, TG, TG*PC, PC*ST, TG*ST, TG*PC*ST

Model 2. BS, ST, PC, TG, TG*PC, PC*ST, TG*ST

Model 3. BS, ST, PC, TG, TG*PC, PC*ST

Model 4. BS, ST, PC, TG, TG*PC

Model 5. BS, ST, PC, TG

In the event of statistically significant interaction terms, TG*PC*ST, TG*ST or PC*ST, the interpretation of the analysis was explored further.

The absence of a statistically significant TG*PC interaction would suggest that prior use of cannabis has no association with the assessment of efficacy. Thus, there would be no evidence to suggest that the treatment difference differs between prior and non-prior cannabis users, that is, even if prior users are able to distinguish between the treatments, this is not likely to have led to bias in the assessment of efficacy.

At all stages, the interactions were to be carefully evaluated to determine whether they are of degree (quantitative) or direction (qualitative), and how they might influence the treatment contrast.

It is noted that these analyses could have been done on the three individual studies. However, the studies were only powered for their primary comparison and would have lacked power for examining the interaction terms that are so important to this methodology. In addition, the issue of blinding was raised in relation to a regulatory submission, by which time the studies had already concluded with no possibility of re-sizing them to provide reasonable power for the assessment of interaction. Consequently, it was decided to take this meta-analytic approach in order to look for systematic evidence of bias and to maximise the power of these comparisons.

The distribution of the residuals from the final model was examined for evidence of departures from the model’s assumptions, and, in particular, the Shapiro-Wilk test was used to assess the normality of the fitted residuals. In the event of gross-departures for the underlying assumptions, the data (response and baseline covariates) were ranked and these transformed data then analysed using the same linear model approach.

### Distribution of adverse events: prior v naïve cannabis users

The patients most likely to be able to distinguish whether they were in the Sativex® or placebo groups were those who had previous exposure to cannabis. This could manifest itself in a different adverse event profile between prior and naïve users in the Sativex® group compared with the placebo group. The following adverse events were listed as potential effects of cannabis on the Information Leaflet given to patients prior to them giving informed consent for participating in the studies:

○ dizziness, disturbance in attention, euphoric mood, disorientation, feeling disconnected, loss of balance, difficulty with speaking, confusion, worry, paranoia, fainting, hallucinations, disorientation, poor concentration and/or forgetfulness, losing touch with reality, a feeling of general happiness or sadness, feeling abnormal or drunk (nervous system disorders)

○ fatigue, tiredness or drowsiness, weakness, increased incidence of falling, lethargy (general disorders and administration site conditions)

○ nausea, dry mouth, diarrhoea, thirst, throat irritation, feeling sick, being sick, abdominal discomfort, increase or decrease in appetite, changes in sense of taste, stinging or discomfort in the mouth and on the tongue (gastrointestinal disorders)

Consequently, events classified using the Medical Dictionary for Regulatory Activities (MedDRA) into system organ classes “nervous system disorders”, “general disorders and administration site conditions”, “gastrointestinal disorders” and “psychiatric disorders” were examined individually where they were reported by at least 10 patients (using the MedDRA preferred terms) in the analysis; events that occurred in fewer than 10 patients were considered to be too rare to provide reliable results.

The number of patients reporting these adverse events, by preferred term, was summarised across the included studies and displayed by treatment group and prior/naïve cannabis use within the treatment group. The odds ratio for patients having received Sativex® (as opposed to placebo) for both prior cannabis users and cannabis naïve users was calculated for each AE together with 95% confidence intervals.

Also, for each of these events, a logistic model was used to assess the odds ratio (Sativex®/Placebo) adjusted for prior cannabis exposure. Fixed factors included in the model were treatment group (Sativex®/placebo) (TG), Prior use of cannabis (Yes/No) (PC), Study (ST), and the interaction terms TG*PC, TG*ST, PC*ST, TG*PC*ST. The TG*PC interaction and PC main effect were to be inspected for evidence of an association between prior cannabis exposure and the adverse event profile, either as an interaction with treatment group or independent of treatment. If the TG*PC interaction was statistically significant then that would provide evidence of possible unblinding. If it were not, but the main effect of PC in the model excluding the TG*PC interaction was statistically significant, this would suggest that more (or fewer) prior cannabis users experienced the event than cannabis naïve patients. But it would not provide evidence that they would be more likely to identify their study treatment. The TG term was to be inspected for evidence of a differential adverse event profile between the treatment groups. Again, if it was statistically significant it would not necessarily provide evidence of unblinding. Odds ratios and 95% CIs were to be presented, as appropriate.

### Association between the experience of central nervous system events and assessment of efficacy

The association of the three most frequently occurring central nervous system adverse events and the assessment of efficacy was investigated using the assessment of spasticity over the primary period during the three studies.

As in the previous analysis, the data were analysed using a general linear model. The dependent variable was the change from baseline in spasticity assessment. Fixed factors included in the model were treatment group (Sativex®/placebo) (TG), experienced one or more of the three AEs (Yes/No) (AE), study (ST), and the interaction terms TG*AE, TG*ST, AE*ST, TG*AE*ST. Baseline spasticity (BS) was included as a covariate in all models. The same stepwise model selection process described above (with the term PC replaced by AE) was completed.

The interpretation of these analyses would be that if the occurrence of one of the three most frequent CNS AEs has no association with the assessment of efficacy, this would be evidenced by no statistically significant TG*AE interaction. Thus, there would be no evidence to suggest that the treatment difference is itself different for patients who had experienced at least one of these events, that is, even if patients experiencing these events realise which treatment they are taking, it is not likely to have led to bias in the assessment of efficacy.

### Dose of test medication: prior v naïve cannabis users

In each of the studies, participants titrated to their own preferred daily dose (number of sprays) of study medication. This was done by titrating the doses until satisfactory symptom relief was obtained or until unwanted side-effects occurred. If prior use of cannabis allowed patients to determine which medication they have been given then it might be expected that their titrated dose would be different from cannabis naïve patients; for example, in the placebo treatment group patients with previous exposure to cannabis might take fewer doses than the cannabis naïve patients if they realised that they were on a placebo (futility), especially given that the alcohol in the excipients is known to cause oral mucosal stinging in some study participants.

A general linear model was used exactly as for prior cannabis use in relation to efficacy with the exceptions that the dependent variable was to be the patients’ mean sprays per day of test medication during the studies and there was no baseline covariate. The interpretation of these analyses would be that if prior cannabis exposure has no association with the sprays of test medication used, as evidenced by no statistically significant TG*PC interaction, then any unblinding as a result of prior exposure to cannabis is not likely to have led to a different dosing pattern between prior exposure to cannabis and naïve patients.

In all of these analyses no formal adjustment was made for the multiplicity involved in considering such a large number of analyses, with a 5% significance level to be used for each individual analysis. This multiplicity needs to be considered when interpreting the above results.

## Results

A total of 666 people with spasticity were randomised and treated in the three studies to form the safety population: 363 to Sativex® and 303 to Placebo. The ITT population comprised 652 patients: 356 on Sativex® and 296 on Placebo. Table 1 shows the breakdown by individual study for the ITT population.

**Table 1:** The ITT populations with and without prior cannabis use in each of the studies

	**Sativex**® **(n = 356)**		**Placebo (n = 296)**		**Total (n = 652)**	
	**PC**	**No PC**	**PC**	**No PC**	**PC**	**No PC**
**Study**						
GWCL0403	33	133	47	122	80	255
GWMS0001	33	37	32	30	65	67
GWMS0106	51	69	27	37	78	106

### Association between prior use of cannabis and assessment of efficacy

Overall, the proportion of patients with prior experience of cannabis was fairly similar between the two treatment groups: 117/356 (33%) in the Sativex® group and 106/296 (36%) in the Placebo group, although there was some variability between studies. Table 1 shows the breakdown by individual study in the ITT population. Overall, the proportion of patients reporting prior use of cannabis (34%) is slightly lower than the 43% reported in the literature [12].

The results of Model 1 using ANOVA showed that the three-way interaction, TG*PC*ST was not statistically significant (*P* = 0.19) and it was removed. Neither of the two-way interactions involving study, TG*ST and PC*ST, were statistically significant (*P* = 0.72, 0.51 and Models 2 and 3, respectively) and so were removed. The model (Model 4) containing baseline spasticity, the main effects ST, PC, TG and the two-way interaction TG*PC showed that the interaction TG*PC was not statistically significant (*P* = 0.63). Model 5, containing only baseline spasticity and the main effects ST, PC and TG, showed that the main effect of prior use of cannabis was not statistically significant overall (*P* = 0.11). These results are summarized in Table 2.

**Table 2:** Analysis of efficacy adjusting for prior use of cannabis

**Source**	***P*-value**				
	**Model 1**	**Model 2**	**Model 3**	**Model 4**	**Model 5**
Baseline severity (BS)					<0.0001
Study (ST)					0.052
Prior cannabis (PC)					0.105
Treatment group (TG)					0.033
TG*PC	0.962	0.637	0.561	0.626	
PC*ST	0.497	0.514	0.495		
TG*ST	0.810	0.720			
TG*PC*ST	0.188				
Estimated effect of previous cannabis use on spasticity NRS (95% CI)	Previous users:			−1.23 (−1.43 to −1.04)	
	No previous use:			−0.98 (−1.22 to −0.74)	
	Estimated difference:			−0.25 (−0.56 to 0.05)	

Summary of ANOVA.

The residuals from the model containing baseline spasticity, the main effects ST, PC, TG and the two-way interaction PC*TG were clearly not Normally distributed (Shapiro-Wilk W test: *P* <0.0001). Accordingly, a nonparametric rank analysis of covariance was performed using the ranks for the baseline spasticity and change from baseline. The results were very similar to the parametric analyses using the observed data – two-way interaction TG*PC was not statistically significant (*P* = 0.37), and the main effect of prior cannabis use was not statistically significant (*P* = 0.16).

In summary, we found no statistical evidence of a relationship between efficacy and prior use of cannabis either overall or between the treatment groups.

### Distribution of adverse events: prior v naïve cannabis users

The analysis of the distribution of AEs between prior cannabis users and cannabis naïve patients used the Safety population. Overall the proportion of patients with prior experience of cannabis was fairly similar between the two treatment groups: 121/363 (33%) in the Sativex® group and 110/303 (36%) in the Placebo group. After consolidating the AEs described, there were 135 different preferred terms (34 in “gastrointestinal disorders”, 23 in “general disorders and administration site conditions”, 45 in “nervous system disorders” and 33 in “psychiatric disorders”). Twenty-five of them occurred in 10 or more patients. For this analysis the most important model is the one comprising ST PC TG TG*PC. For the 13 AEs where the number of patients experiencing the event was 20 or more, this model always converged. When the number of patients experiencing a particular AE was less than 20, sometimes this model did not converge but the model containing only the main effects ST PC TG did converge, and sometimes neither model converged.

Table 3 summarises the number of patients reporting these 25 most common AEs, by the preferred term, across the included studies and displayed by treatment group and prior/naïve cannabis use within each treatment group. This table is ordered by system organ class. The odds ratio for patients having received Sativex® (as opposed to placebo) for both prior cannabis users and cannabis naïve patients are presented for each AE together with 95% confidence intervals.

**Table 3:** AEs by treatment and prior experience of cannabis (where n >/= 10)

**Body system**	**Preferred term**		**Sativex®**	**Placebo**	**OR (95% CI)**
	**n**		**n (%)**	**n (%)**	
Nervous system disorders	Dizziness	PC	32 (26%)	13 (12%)	2.7 (1.3 to 5.4)
	n = 150	No PC	84 (35%)	21 (11%)	4.33 (2.6 to 7.3)
	Headache	PC	9 (7%)	14 (13%)	0.55 (0.2 to 1.3)
	n = 57	No PC	21 (9%)	13 (7%)	1.31 (0.6 to 2.7)
	Somnolence	PC	17 (14%)	3 (3%)	5.8 (1.7 to 20.5)
	n = 45	No PC	19 (8%)	6 (3%)	2.6 (1.0 to 6.7)
	Muscle spasticity	PC	2 (2%)	2 (2%)	0.9 (0.1 to 6.6)
	n= 33	No PC	17 (7%)	12 (6.3%)	1.1 (0.5 to 2.4)
	Balance disorder	PC	6 (5%)	3 (2.7%)	1.9 (0.5 to 7.6)
	n = 20	No PC	8 (3%)	3 (2%)	2.2 (0.6 to 8.2)
	Disturbance in attention	PC	7 (6%)	0	
	n = 19	No PC	12 (5%)	0	
	Dysgeusia	PC	3 (3%)	1 (1%)	2.8 (0.3 to 27.0)
	n= 18	No PC	13 (5%)	1 (0.5%)	10.8 (1.4 to 83.6)
	MS relapse	PC	0	2 (1.8%)	
	n = 17	No PC	8 (3%)	7 (4%)	0.9 (0.3 to 2.5)
	Dysarthria	PC	5 (4%)	0	
	n = 13	No PC	2 (3%)	2 (1%)	2.4 (0.5 to 12.1)
Psychiatric disorders	Disorientation	PC	9 (7%)	1 (1%)	8.8 (1.1 to 70.3)
	n = 21	No PC	10 (4%)	1 (0.5%)	8.2 (1.0 to 64.9)
	Depression	PC	5 (4%)	3 (3%)	1.5 (0.4 to 6.6)
	n = 19	No PC	8 (3%)	3 (2%)	2.2 (0.6 to 8.2)
	Euphoria	PC	3 (3%)	3 (3%)	0.9 (0.2 to 4.6)
	n = 13	No PC	4 (2%)	3 (2%)	1.1 (0.2 to 4.8)
	Insomnia	PC	0	1 (1%)	
	n = 10	No PC	4 (2%)	5 (3%)	0.6 (0.2 to 2.4)
General disorders	Fatigue	PC	17 (14%)	9 (8%)	1.8 (0.8 to 4.3)
	n = 107	No PC	50 (21%)	31 (17%)	1.4 (0.8 to 2.2)
	Application site pain	PC	6 (5%)	6 (6%)	0.9 (0.3 to 2.9)
	n = 34	No PC	12 (5%)	10 (5%)	0.9 (0.4 to 2.2)
	Asthenia	PC	9 (7%)	7 (6%)	1.2 (0.4 to 3.3)
	n = 45	No PC	22 (9%)	7 (4%)	2.6 (1.0 to 6.8)
	Feeling abnormal	PC	5 (4%)	1 (1%)	4.7 (0.5 to 40.9)
	n = 12	No PC	5 (2%)	1 (1%)	4 (0.5 to 34.8)
	Feeling drunk	PC	3 (3%)	0	
	N = 11	No PC	8 (3%)	0	
GastroIntestinal Disorders	Oral discomfort	PC	12 (10%)	10 (9%)	1.1 (0.5 to 2.7)
	n = 45	No PC	13 (5%)	10 (5%)	1.0 (0.4 to 2.4)
	Nausea	PC	12 (10%)	8 (7%)	1.4 (0.6 to 3.6)
	n = 67	No PC	32 (13%)	15 (8%)	1.8 (0.9 to 3.4)
	Dry mouth	PC	6 (5%)	6 (6%)	0.9 (0.3 to 2.9)
	n = 37	No PC	21 (9%)	4 (2%)	4.5 (1.5 to 13.2)
	Diarrhoea	PC	8 (7%)	7 (6%)	1.0 (0.4 to 3.0)
	n = 30	No PC	12 (5%)	3 (2%)	3.3 (0.9 to 11.8)
	Vomiting	PC	2 (2%)	1 (1%)	1.8 (0.2 to 20.5)
	n = 16	No PC	7 (3%)	6 (3%)	0.9 (0.3 to 2.8)
	Constipation	PC	5 (4%)	3 (3%)	1.5 (0.4 to 6.6)
	n = 15	No PC	7 (3%)	0	
	Dyspepsia	PC	1 (1%)	2 (2%)	0.5 (0.0 to 5.0)
	n = 14	No PC	7 (3%)	4 (2%)	1.4 (0.4 to 4.9)

For none of these 25 most common AEs were the three-way TG*PC*ST interaction or either of the two-way PC*ST and TG*ST interactions statistically significant at the 5% level. The TG*PC interaction was not significant for any AE. When the TG*PC interaction was removed and the model contained only the main effects, there was only one AE where the effect of PC was statistically significant at the nominal 5% level (Somnolence: *P* = 0.017).

In summary, there was no significant relationship between any AE (apart from potentially somnolence) and prior use of cannabis, and there is no suggestion of a different influence of prior cannabis use between the active and placebo groups.

### Association between central nervous system events and the assessment of efficacy

The three most common nervous system disorder AEs were dizziness, headache and somnolence, occurring in 150 (23%), 57 (9%), 45 (7%) of patients, respectively. There was a notably increased risk of dizziness on Sativex® over placebo for each of the three studies, as well as overall. Overall, somnolence was also more likely to be experienced by patients on Sativex®, whilst there was no evidence of a difference between the treatment groups in the likelihood of experiencing headache either for the individual studies or overall. Taking the three events together, then overall patients on Sativex® were more likely to experience at least one of these events than those on placebo.

The modelling (Model 1) showed that the three-way interaction, TG*AE*ST, was not statistically significant (*P* = 0.69) and it was removed. The two-way interaction TG*ST was also not statistically significant (*P* = 0.79, Model 2) and was removed. In the model containing the two remaining two-way interactions (Model 3) AE*ST was statistically significant (*P* = 0.01) while TG*AE was not (*P* = 0.13). So next, the two-way interaction TG*AE was removed (Model 4); the final model contained baseline spasticity, the main effects ST, AE, TG and the statistically significant two-way interaction AE*ST (*P* = 0.017). Table 4 shows a brief summary of the analysis of these models.

**Table 4:** Analysis of Efficacy adjusting for the three most frequent Nervous System Disorder AEs – summary of ANOVA

	***P*-values**			
**Source**	**Model 1**	**Model 2**	**Model 3**	**Model 4**
Baseline severity (BS)				<0.0001
Study (ST)				0.15
Adverse event (AE)				0.49
Treatment group (TG)				0.033
AE*ST	0.008	0.011	0.010	0.017
TG*AE	0.15	0.13	0.13	
TG*ST	0.80	0.79		
TG*AE*ST	0.69			
Estimated effect of AE occurrence on spasticity NRS (95% CI)	Previous users:		−1.08 (−1.35 to −0.82)	
	No previous use:		−1.20 (−1.38 to −1.01)	
	Estimated difference:		−0.11 (−0.44 to 0.21)	

In study GWCL0403, there was no marked difference between the two AE groups. In study GWMS0001, there appeared to a greater reduction in spasticity in the groups experiencing none of the AEs, while in study GWMS0106, the greater reduction in spasticity appeared to be in the group which experienced one or more of the AEs. These differences were statistically significant (*P* = 0.017). There was no evidence of a difference in the predicted means for the treatment group by AEs cross-classification (*P* = 0.13).

The residuals from the model containing baseline spasticity, the main effects ST, AE, TG and the two-way interaction ST*AE were clearly not Normally distributed (Shapiro-Wilk W test: *P* <0.0001). Accordingly, a nonparametric rank analysis of covariance was performed using the ranks for the baseline spasticity and change from baseline. The results were a little different from the analyses using the observed data; two-way interaction ST*AE was not statistically significant in the model containing AE*ST and TG*AE (*P* = 0.12). When AE*ST was removed, TG*AE was not statistically significant (*P* = 0.26) and when this interaction was removed, the main effect AE was not statistically significant (*P* = 0.87).

In summary, there is no evidence of a relationship between overall treatment effect and experience of one or more of these three AEs. There was some statistical evidence, when using the regular ANCOVA, that the relationship between efficacy and experience of one of these AEs was different between the studies but in different directions in different studies; a supporting non-parametric analysis did not confirm the presence of a relationship between efficacy and the study by AE experience interaction. So, overall there is no evidence that experiencing one or more of these AEs is associated with the change from baseline in spasticity in any consistent way.

### Dose of test medication: prior v naïve cannabis users

Table 5 shows the mean sprays per day of test medication by treatment group, study and prior use of cannabis. Table 6 shows a brief summary of the models fitted to these data. Model 1 showed that the three-way interaction, TG*PC*ST, was not statistically significant (*P* = 0.23) and it was removed. The two-way interaction, PC*ST (Model 2), was also not statistically significant (*P* = 0.90) and was removed. In the model containing the two remaining two-way interactions, (Model 3) ST*PC and TG*PC, ST*PC was close to being statistically significant (*P* = 0.058) while TG*PC was not (*P* = 0.64). After TG*ST was removed TG*PC was not significant (*P* = 0.77, Model 4) and was removed. In the model with only main effects (Model 5), PC was not significant (*P* = 0.48) and was removed. In both treatment groups; there was little difference between the previous users and the cannabis naïve patients.

**Table 5:** Mean number of daily doses taken by patients in the three clinical studies according to their previous experience of cannabis

**Study**		**Prior Cannabis**	**n**	**Mean daily doses**	**SD**
GWCL0403	Sativex®	PC	33	8.47	5.06
		No PC	133	8.49	4.70
	Placebo	PC	47	14.69	6.34
		No PC	122	15.67	6.08
GWMS0001	Sativex®	PC	33	13.78	10.2
		No PC	37	13.27	7.66
	Placebo	PC	32	21.72	11.15
		No PC	30	24.75	10.83
GWMS0106	Sativex®	PC	51	8.97	7.04
		No PC	69	9.79	6.02
	Placebo	PC	27	15.83	9.37
		No PC	37	14.70	7.59

**Table 6:** Relationship between prior use of cannabis and study drug dosing; summary of ANOVA

**Source**	**Model 1**	**Model 2**	**Model 3**	**Model 4**	**Model 5**
Baseline severity (BS)					0.18
Study (ST)					<0.0001
Prior cannabis (PC)					0.48
Treatment group (TG					<0.0001
TG*PC	0.66	0.70	0.64	0.77	
TG*ST	0.07	0.06	0.06		
PC*ST	0.72	0.89			
TG*PC*ST	0.23				
Estimated effect of previous cannabis use on dosing (95% CI)	Previous users:			13.9 (13.0 to 14.9)	
	No previous use:			14.4 (13.6 to 15.1)	
	Estimated difference:			0.43 (−0.76 to 1.61)	

The residuals from the model containing baseline spasticity, the main effects ST, PC, TG and the two-way interaction TG*ST, were clearly not Normally distributed (Shapiro-Wilk W test: *P* <0.0001). Accordingly, a nonparametric rank analysis of covariance was performed using the ranks for the baseline spasticity and change from baseline. The results were fairly similar to the analyses using the observed data, except that the two-way interaction TG*ST was clearly not statistically significant (*P* = 0.30). In summary, no interaction involving prior use of cannabis was statistically significant, nor was the main effect when the interactions were removed. This suggests that the dosing of study medication was not affected by prior use of cannabis.

## Discussion

The results of our analysis show that neither prior experience of cannabis, nor the occurrence of the most common CNS adverse events has a significant effect on the change in subjective patient-reported spasticity severity score in a large cohort of patients with multiple sclerosis treated with Sativex®. This allows for the conclusion that there is no evidence that any unblinding that may have occurred in these studies was likely to have affected the outcome. This is an important contribution to confirming the internal validity and scientific integrity of the three randomized controlled studies which contributed to our analysis.

In a controlled, double-blind clinical study, unblinding of patients to treatment allocation has the potential to introduce bias into the results, most notably where the outcome measures are subjective [4]. The most appropriate means by which to assess whether patients have become unblinded to treatment allocation is the subject of some debate. The most frequently used approach is simply to ask patients to guess which treatment they are taking, and to describe and assess the statistical significance of the results. Indeed, this approach has been strongly endorsed. However, there is no accepted best practice on how to ask the question, nor how to analyse the results [17,18].

While the most commonly employed approach is to ask the patient at the end of the study, other approaches ask at the beginning of the study, or both at the beginning and the end. Some methods permit a ‘don’t know’ response, while others do not. The statistical methodology used to assess the significance of the guesses also varies, with a Chi square or Fisher’s exact test being the most frequently employed [17]. Alternatively, James *et al.*[19] and, subsequently, Bang *et al.*[20] have proposed a ‘blinding index’, which aims to provide a more systematic and consistent way of dealing with guesses regarding treatment allocation. In one published study of a different cannabis-based medicine than has been investigated in this report, patients were asked to guess their treatment allocation, and there was evidence that they did so to a greater degree than expected [21]. In that study, the rate of nervous system adverse events was also substantially higher than was seen in the three studies we report. While the authors of that study interpreted the results of the treatment guesses as an indication that patients were unblinded, we would propose that it might have been a reflection of greater efficacy. A systematic study of the impact of unblinding on patients’ and physicians’ judgement of the effect of a treatment on multiple sclerosis was published in a classic paper by Noseworthy *et al.*[22]. That study suggested that the impact of being unblinded to treatment was greater when the physician was unblinded than when the patient was unblinded.

In all these cases, the fundamental problem of how to interpret the patients’ guesses remains; a correct guess may be a surrogate for the efficacy of a medicine with a subjective outcome measure, or an indication that the medicine has a characteristic adverse event profile. Indeed, it is questionable whether such data can provide valid information about possible unblinding [7,8].

In the analysis that we report here, there was little evidence that experience of any of the three most common nervous system adverse events was associated with the change from baseline in the patient-reported severity of spasticity. If the patients had become unblinded by the adverse event, then it would be expected that such unblinding may have led to an overestimation by the patient of the efficacy, since they would be more likely to believe that they were on active treatment. Similarly, efficacy was no greater in those patients who reported prior use of cannabis; were the patients able to ‘recognise’ the cannabis-based medicine because of prior experience of cannabis, then it might be expected that they would overestimate the effects of treatment – and they did not. The observation that patients with prior experience of cannabis did not show a different dosing pattern of study medication helps confirm that they were unlikely to have been unblinded as a result of their prior experience.

The approach we have adopted has limitations. For example, it is feasible that even if patients do become aware of their treatment allocation, this might not affect their assessment of the impact of the medicine on their condition. Or even that awareness of their treatment allocation may predispose them to lack of efficacy or to report particular adverse events, depending on their expectation of the effects of the active treatment. For this reason, we believe that the kind of analysis that we have done here is better designed to determine not so much whether unblinding is likely to have occurred, but whether any unblinding that may have occurred is likely to have had an impact on the outcome. Our approach also assumes that patients who become unblinded to treatment allocation are likely to express bias in the way they assess the efficacy of the treatment in a similar way. It remains possible that sub-groups of patients may have become unblinded to treatment allocation, and that this unblinding may have affected their assessment of the efficacy of the treatment in opposite directions, thus resulting in no overall impact of unblinding. It is not possible to detect whether this may have occurred.

This discussion also raises questions about what really constitutes effectiveness. In the setting of clinical medicine, the patient is of course always aware of their treatment allocation, and yet the prescriber is generally willing to accept their report of the effectiveness of the treatment. It is perhaps paradoxical that we are prepared to accept the patient report in the setting of the therapeutics of a condition in clinical practice, but not in the setting of a clinical trial.

Despite this, there is little doubt that maintaining the integrity of the blind in a double-blind study contributes to the internal validity of the study, even if the external validity may be limited. Equally, it seems clear that the most commonly used methodology for investigating the maintenance of the blind is limited. Therefore, there exists a need for alternative approaches to investigating this source of potential bias in the setting of the randomized controlled clinical study.

Because of this, we took what we believe to be the more rigorous approach whereby we first identified the likely factors that might lead to unblinding, and then systematically investigated the impact of those factors on the subjective outcome of the studies. This approach avoids the paradox described by Fisher in his definitive essay ‘Mathematics of a Lady Tasting Tea’ [23], whereby the fact that the study participants correctly guess their treatment allocation reflects the difference between treatments, and is regarded as invalidating the study, and at the same time validates the study hypothesis that the treatments are indeed distinguishable [5]. Our approach also concentrates on the impact that possible unblinding has on the study outcome, rather than describing the patients’ views on the treatment that they received. In this way, our methodology adds directly to the credibility of the outcome. We believe that this methodology can be applied to studies of a variety of study medications, particularly where there is a subjective primary outcome measure, and where the drug has a distinct adverse event profile.

## Conclusions

We are able to conclude from this investigation that the presence of factors which might be assumed to lead to unblinding of some patients to their treatment allocation, had no significant impact on the subjective efficacy endpoint used in three separate placebo-controlled clinical trials of a cannabis-based medicine. This suggests not only that widespread unblinding was unlikely, but also that what unblinding may have occurred did not introduce bias into the assessment of the efficacy of Sativex® in the relief of spasticity due to multiple sclerosis. We propose that this methodology is suitable for the investigation of potential bias of other medicines with a characteristic adverse event profile, and where efficacy is assessed using subjective measures.

## Abbreviations

AE: Adverse Event; ANOVA: Analysis of Variance; BS: Baseline spasticity; CBD: Cannabidiol; CNS: Central Nervous System; ITT: Intent-to-Treat; MedDRA: Medical Dictionary for Regulatory Activities; NRS: Numeric Rating Scale; PC: Prior cannabis user; ST: Study; TG: Treatment Group; THC: Delta-9-tetrahydrocannabinol.

## Competing interests

SW and PD are full-time employees of GW Pharma Ltd.

DA acted as a Consultant to GW Pharma in the design execution and interpretation of these analyses.

## Authors’ contributions

All authors contributed actively at all stages of the planning, execution and writing of this piece of research. All authors read and approved the final manuscript.
